# Psychosocial factors associated with early initiation and frequency of antenatal care (ANC) visits in a rural and urban setting in South Africa: a cross-sectional survey

**DOI:** 10.1186/s12884-016-0807-1

**Published:** 2016-01-25

**Authors:** Lorrein Shamiso Muhwava, Neo Morojele, Leslie London

**Affiliations:** School of Public Health and Family Medicine, University of Cape Town, Cape Town, South Africa; Medical Research Council, Pretoria, South Africa; School of Public Health, University of the Witwatersrand, Johannesburg, South Africa

**Keywords:** Maternal Mortality, Antenatal care, Psychosocial factors, Rural, Urban, South Africa

## Abstract

**Background:**

Late booking and infrequent antenatal care (ANC) are common but avoidable patient-related risk factors for maternal deaths in South Africa. The aim of the study was to examine the association of psychosocial factors with early initiation of ANC and adequate frequency of attendance of ANC clinics among women in an urban and rural location in South Africa.

**Methods:**

Data from a 2006 cross-sectional household survey of 363 women from the rural Western Cape and 466 women from urban Gauteng provinces of South Africa for risk of alcohol-exposed pregnancy were analysed. We examined associations between psychosocial variables (self-esteem, cultural influences, religiosity, social capital, social support, pregnancy desire (wanted versus unwanted pregnancy), partner characteristics and mental health) and both early ANC first visit (before 16 weeks) and adequate frequency of ANC visits (4 or more visits) for respondents’ last pregnancy.

**Results:**

Overall prevalence among urban women of early ANC initiation was 46 % and 84 % for adequate ANC frequency. Overall prevalence among rural women of early ANC initiation was 45 % and 78 % for adequate ANC frequency. After adjusting for clustering, psychosocial factors associated with early ANC initiation in the urban site were being employed (OR 1.6; 95 % CI 1.0–2.5) and wanted pregnancy (OR 1.8; 95 % CI 1.1–3.0). For the rural site, early ANC initiation was significantly associated with being married (OR 1.93; 95 % CI 1.0–3.6) but inversely associated with high religiosity (OR 0.5; 95 % CI 0.3–0.8). Adequate frequency of ANC attendance in the rural site was associated with wanted pregnancy (OR 4.2; 95 % CI 1.9–9.3) and the father of the child being present in the respondent’s life (OR 3.0; 95 % CI 1.0–9.0) but inversely associated with having a previous miscarriage (OR 0.4; 95 % CI 0.2–0.8). There were no significant associations between adequate ANC attendance and the psychosocial factors in the urban site.

**Conclusion:**

The majority of women from both sites attended ANC frequently but less than 50 % initiated ANC before the recommended 16 weeks gestational age. Interventions to reduce prevalence of late ANC booking and inadequate ANC attendance should engage religious leaders, address unintended pregnancy through family planning education and involve male partners in women’s reproductive health.

**Electronic supplementary material:**

The online version of this article (doi:10.1186/s12884-016-0807-1) contains supplementary material, which is available to authorized users.

## Background

South Africa is amongst countries with the highest rates of maternal and perinatal mortality globally [1]. The South African Confidential Enquiries into Maternal Deaths for 2008–2010 found an institutional maternal mortality ratio (MMR) of 176.22/100000 live births in 2008–2010 compared to 151.77/100000 live births in 2005–2007 and that the institutional MMR is still increasing [2]. According to the World Health Organization (WHO) [3], the rate of decline of MMR in sub-Saharan Africa has been less than 1 % per year and, in fact, the ratios of maternal mortality have increased in countries such as South Africa, Nigeria, Mozambique and Swaziland. Infrequent, poor and no antenatal care, as well as delay in accessing medical help were listed in The South African Confidential Enquiries into Maternal Deaths for 2008–2010 [2] as amongst the most frequent patient-related avoidable factors resulting in maternal deaths, representing important missed opportunities for prevention.

The WHO recommends four goal-oriented visits, known as the focused ANC package, as an adequate and effective number of visits for the provision of essential interventions for pregnant women with no underlying health conditions, with the first visit occurring in the first trimester [4, 5]. In resource limited settings, increasing the number of antenatal care visits to more than four has not been found to improve health outcomes in uncomplicated pregnancies [6]. On the other hand, in low and middle income countries (LMIC), attendance of less than four ANC visits has been associated with an increased risk of perinatal mortality, particularly, stillbirth [7].

Although an ANC visit within the first 16 weeks is recommended in most country guidelines, this has not translated into practice amongst women in South Africa and late booking, despite the recommended guidelines, remains the trend in most countries in sub-Saharan Africa [8–10]. According to the Saving Mothers Report (2005–2007) [2] and the Millennium Development Goals 2009 Report [11], whilst over 90 % of South African women have access to antenatal care services, only 63.2 % of pregnant women in South Africa attend antenatal services at all.

Previous studies have identified demographic factors, physical access to health facilities, parity, lack of health education, relationships with health care providers and misconceptions of antenatal care (ANC) as factors influencing timing of ANC booking [10, 12, 13].

Psychosocial epidemiology as described in [14] is concerned with the interactions people have with their environent and the influence of these interactions on their health – whether directly or indirectly. There is no consensus in literature on which theories best explain psychosocial concepts. The focus of the relationship between psychosocial factors and timing of initiation of antenatal care has mainly been around three factors; overall stress level and coping strategies, risk-taking behaviour and feelings about the pregnancy [15] and the research on the associations between psychosocial factors and antenatal care attendance is also currently quite limited and fairly new [7, 13, 16].

The aim of the study was to examine the association between psychosocial factors and ANC booking to determine whether psychosocial factors were associated with not only timing of initiation of antenatal care but also with frequency of attendance of ANC clinics amongst women in an urban and rural location in South Africa reporting on their last pregnancy. Identifying these psychosocial factors would be useful for making decisions on holistic interventions focusing not only on the clinical aspects of pregnancy but the psychosocial wellbeing of the woman and also health education programmes targeted at improving health outcomes for pregnant women and the unborn child in South Africa.

## Methods

### Sampling method and sample size

The data was derived from a 2006 cross-sectional household survey which assessed and compared the predictors of risk of alcohol-exposed pregnancies among women (18–44 years old) from two contrasting sites; a rural and urban site in South Africa [17]. Participants were recruited from an urban area (*N* = 606) located in the Tshwane Metropolitan Municipality in the Gauteng region and a rural area (*N* = 412) in the West Coast district of the Western Cape. The two provinces are broadly similar in level of socio-economic wealth, age/gender structure and disease burden outcome and the different sites were chosen primarily for urban/rural difference and for differences in race composition. Cluster random sampling was used at the urban site with a target sample size of 820 women. Random selection was used to select 82 census enumeration areas (EA) out of 450 areas; from each EA, 10 households were then selected using aerial photographs. One woman was selected randomly within each household, provided she met the eligibility criteria after a brief household census identified all adults in the housheold 18 years or older. Stratified cluster random sampling was used at the rural site with a target sample size of 650 women. Farms were chosen within the boundaries of the three municipal areas. Out of 1450 farms listed, 150 farms were randomly selected. All eligible women on each farm were approached to participate in the study to improve recruitment as there were generally a small number of households per farm (approximately 7).

Trained fieldworkers conducted face-to-face interviews using structured questionnaires in the participant’s preferred language from a choice of five common languages among the study population. The questionnaire contained measures which have largely been used previously in similar populations in South Africa. The interviews took between 15 and 90 min and were conducted in the privacy of the participant’s home. In total, 606 women were interviewed in urban site and 412 women were interviewed in the rural site corresponding to response rates of 74 % and 83 % respectively [17].

**Fig. 1:**
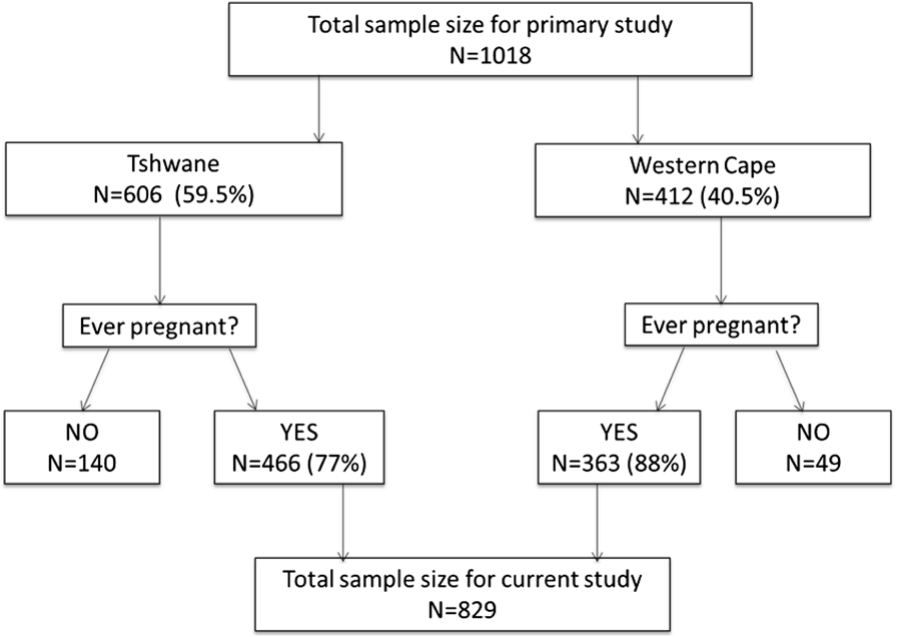
Sample selection of women who met the inclusion criteria from the 2006 cross-sectional household survey. From the participants of the primary study, women who had neither given birth in their life time nor had a miscarriage were excluded from this study. Women who reported being currently pregnant were only eligible for the study provided this was not their first pregnancy

For the purposes of this study, only women who had ever been pregnant were eligible and therefore data of women who had never been pregnant ie; never given birth or had never had a miscarriage were excluded. Women who reported being currently pregnant were only included in the study provided this was not their first pregnancy i.e., this study focused only on information about the pregnancy of their last born child and not their current pregnancy. A total of 363 women (13 of whom were also currently pregnant) from the rural sample and 466 women (14 of whom were also currently pregnant) from the urban site met the inclusion criteria for the current study (Fig. 1).

### Ethics

In the primary study, the field workers explained the study purpose and participant requirements before obtaining written informed consent from participants during the interview process. Participants were made aware that their participation in the study was entirely voluntary and informed of their right to withdraw their consent before, during or at the end of the interview without consequences. Participants were also given the opportunity to read through the questionnaire and ask questions prior to the interviews. This study is based on data derived from interviews conducted in the primary study. Ethical approval for the primary study was granted by the Faculty of Health Sciences Research Ethics Committees of the Universities of Pretoria (121/2005) and Cape Town (381/2005).

### Dependent variables

We examined two dependent variables (a) timing of ANC (early versus late ANC initiation) and (b) frequency of ANC attendance (adequate versus inadequate ANC attendance) during last pregnancy.

Early initiation (as recommended in literature guidelines) [2, 4] was defined as a first ANC visit at or earlier than 16 weeks during the last pregnancy. The original questionnaire included questions on antenatal care relating to the women’s pregnancy with their last born child. The women were asked their gestational age when they first initiated antenatal care during that (their last) pregnancy. They were asked to report on the number of months pregnant at the time of their first ANC visit, which was easier for the women to recall, and then converted to weeks for analysis. The responses were then coded as either early ANC initiation (1) for those who attended at or before 16 weeks gestational age or late ANC initiation (0) for those who attended after 16 weeks gestational age.

Adequate number of ANC visits was defined as at least four visits during the last pregnancy. This latter criterion follows recommendations by the WHO in the focused ANC model for low resource countries and the recommendations of the basic antenatal care (BANC) approach [5]. Even though the 2007 national Guidelines for Maternity Care in South Africa are not consistent with the WHO guidline (recommend four follow-up visits after the first ANC visit scheduled for 20, 26, 32, and 38 weeks, and 41 if still pregnant for uncomplicated pregnancies – i.e. total 5 or 6 visits), this study used 4 visits as the cut-off for adequate attendance.

Participants responded to the question, “How many times did you go for antenatal appointments during this pregnancy?” The responses were then coded as either Adequate ANC attendance(1) for those who attended at least 4 ANC visits during the pregnancy or Inadequate ANC attendance (0) for those who attended less than 4 ANC visits during the previous pregnancy.

### Independent variables: psycho-social factors

#### Self-esteem

The Rosenburg self-esteem scale is the most widely used self-esteem scale internationally and has been used in studies in South Africa [18–20]. Participants responded to questions of this scale about their general feelings about themselves. Scores on this 10 item self-esteem scale were summed and dichotomised across the 75^th^ percentile to represent low (0) versus high self-esteem (1) [18].

#### Cultural influences

Four one- item scales were used to assess the extent of the participants’ agreement to statements on fertility norms: male entitlement and cultural prescriptions on child bearing obligation to women. A score of “1” was assigned for the response strongly or moderately agree and all other responses were given a score of “0” for each item as a separate variable. These are original items that were developed specifically for the purposes of this study.

#### Religiosity

A 6-item scale was used to ask questions on religious orientation [21]. Responses were summed and dichotomised as high religiosity (1) and low religiosity (0) split at the 75th percentile.

#### Social capital

Scores on a 6 item social capital scale [22] were summed and dichotomised across the 75^th^ percentile into weak social capital (0) versus strong social capital (1).

#### Social support

A 14-item scale from the Rand Medical Outcomes (MOS) Social Support Survey [23] was used to measure social support (excluding Tangible support scale items). Questions relating to emotional support (8 questions); affectional support (3 questions) and positive social interaction (3 questions) were measured and scored. The scores were summed and dichotomised as weak (0) and strong (1) across the 75^th^ percentile. The Rand Medical Outcomes (MOS) Social Support Survey Scale is a globally validated tool and has been used successfully in studies among South African populations [24, 25].

#### Desire to become pregnant

Participants were categorised into two groups based on their responses to a question on their actual desire to become pregnant at the time they became pregnant as ‘Not at all’ (0) or having any desire to become pregant (i.e. ‘not much’, ‘a little’ or ‘a great deal’ as (1). In this study we used ‘pregnancy desire’ to describe whether the pregnancy was wanted to any degree versus unwanted.

#### Partner characteristics

Questions relating to the participant’s male partner included whether the male partner who was present in her life during the previous pregnancy was the father of the child she was carrying; and the male partner’s age, level of education, and employment status.

#### Mental health

The MOS Short-Form-General Health Survey is an accepted standardized tool used to measure mental health status and has been used in several studies in South Africa which demonstrated its validity [24, 26]. Mental health was assessed by asking participants a series of 5 questions about the way they had been feeling during the past month with responses ranging from ‘All the time’ given a score of “0” to ‘None of the time’ given a score of “6” [27]. The scores were summed and whilst a score of 100 indicated best possible functioning, a cut-off score of 67 or lower indicated low mental functioning (0) and all others above 67 assigned (1).

#### Substance use

For substance use, lifetime smoking was coded (1) for women who had ever smoked in their lifetime and (0) for those who had never smoked. Similarly, lifetime alcohol use was coded (1) for women who had had a drink containing alcohol in their lifetime and (0) for those who had not. In addition, the Alcohol Use Disorders Identification Test (AUDIT) was used to identify high-risk drinkers with a score of 8 or more indicating high-risk drinking (1) whilst a score of less than 8 indicated low-risk drinking (0). The AUDIT tool, developed by the WHO has been validated internationally to assess hazardous drinking and has been used with good validity in studies in South Africa [28–30].

#### Data analysis

The data was analysed in STATA v12.1 using Pearson’s chi-square test to describe associations between categorical variables for each site. Bivariate and multivariate logistic regression analyses were used to test for associations between the outcome variables: (i) timing of ANC initiation: before 16 weeks (early) versus after 16 weeks (late) gestational age and (ii) frequency of attendance: at least 4 visits (adequate) versus less than 4 visits (inadequate) against the psychosocial variables (substance use, feelings about pregnancy, social capital, social support, cultural beliefs, mental health perceptions, self-esteem and partner characteristics).

Multiple logistic regressions were used to model the effects of the psychosocial variables on the outcome variables. A predictive modelling strategy was used as follows: Variables other than psychosocial variables that were potentially associated with ANC attendance such as demographic factors (age, level of education, marital status, employment status and race), parity and previous miscarriage, were selected by looking at the reduction in the deviance due to adding each potential ‘confounder’ to a model with only the outcome variable to form a baseline model with all relevant ‘confounders’.

Psychosocial variables were then added one at a time to the baseline model and their significance was assessed by looking at the reduction in the deviance. Interactions between the independent variables and confounders were assessed for any change in the effect on the outcome variable. The models were then compared using likelihood ratio chi-square statistics and the model with the lowest Aikakes Information criterion (AIC) was selected as the best model. The urban and rural sites were treated as separate strata in analysis rather than combined in one multivariate analysis because previous research had suggested substantial cultural and social differences in these two populations [17]. To adjust for clustering at the rural site, the multivariate analysis was repeated using the survey command ‘svy’ in STATA. Modeling was done separately for the urban and rural sites. Two final models were therefore selected per site, for (i) timing of ANC initiation and (ii) frequency of ANC attendance for the urban and rural sites.

## Results

### Descriptive statistics

The urban site was made up of 87 % (378/466) black women and 13 % (56/466) coloured women. The rural site was made up of 92 % (329/363) coloured women and 8 % (30/363) black women. In the urban site, 9 % (40/466) compared to 28 %(109/363) in the rural site of the women had completed at least Grade 9 education.

For the urban site, overall prevalence of early ANC initiation was 46 % (216/466) with 84 % (393/466) of the women attending ANC visits adequately. For the rural site, overall prevalence of early ANC initiation was 45 % (165/363) with 78 % (284/363) of the women attending ANC visits adequately.

Additional file 1: Table S1 and Additional file 2: Table S2 report results of logistic regression analyses for bivariate associations of variables that were both significant and non-significant with either early initiation or adequate ANC attendance, respectively.

#### Multivariate analyses

- *Timing of ANC initiation*
  *Rural site*
  Married women were more likely to initiate ANC early compared to unmarried women (OR = 1.9; 95%CI 1.0–3.6) (Table 1). High religiosity was inversely associated early ANC initiation (OR = 0.5; 95%CI 0.3–0.8). Having a partner with higher education (completed above Grade 9) increased the odds of early ANC initiation (OR = 1.9; 95%CI 1.2–3.0) and women with a previous miscarriage were more likely to initiate ANC early (OR = 1.2; 95%CI 0.7–2.3) although the association was not significant.
  *Urban*
  Women who were employed were more likely to initiate ANC early compared to women who were unemployed (OR = 1.6 95%CI 1.0–2.5). Wanted pregnancy was associated with increased odds of early ANC initiation compared to unwanted pregnancy OR = 1.8 95%CI 1.1–3.0).
- *Frequency of ANC attendance*
  *Rural*
  Wanted pregnancy was associated with increased odds of adequate attendance of ANC visits compared to unwanted pregnancy (OR = 4.2 95%CI 1.9–9.3). Having the father of the child she was carrying present in her life during the previous pregnancy was strongly associated with adequate ANC attendance (OR = 3.1 95%CI 1.0–9.0). Having a previous miscarriage was inversely associated with adequate attendance of ANC visits compared to women who had never experienced a miscarriage (OR = 0.4; 95%CI 0.2–0.8). Married women were more likely to adequately attend ANC visits compared to unmarried women (OR = 2.1 95%CI 0.9–4.6) although this association was non-signficant (Table 2).

**Table 1:** Factors associated with early ANC initiation (<16 weeks) among women aged 18–44 years in last pregnancy (*N* = 829)

Variables	Rural *(N = 363)*		Urban *(N = 466)*	
	Odds Ratio^a^ (95 % CI)	*p*-value	Odds Ratio^a^ (95 % CI)	*p*-value
Married	1.9 (1.0–3.6)	0.039*	1.5 (0.9–2.4)	0.131
Previous miscarriage	1.2 (0.7–2.3)	0.501	1.3 (0.7–2.4)	0.330
Employed	-	-	1.6 (1.0–2.5)	0.039*
More than one child	-	-	1.0 (0.6–1.7)	0.859
Wanted pregnancy	-	-	1.8 (1.1–3.0)	0.029*
High Religiosity	0.5 (0.3–0.8)	0.006**	1.3 (0.8–1.9)	0.292
Partner with higher education	1.9 (1.2–3.0)	0.009**	0.6 (0.3–1.1)	0.076
Older Partner Age	-	-	2.4 (0.7–7.7)	0.165
Father of child present	-	-	1.0 (0.3–3.0)	0.933

**p < 0.01 *p < 0.05

^a^Odds ratio adjusted for clustering. Final model for Rural (WC) site: marital status; previous miscarriage, religiosity and partner education. Final model for Urban (Tshwane) site: marital status; previous miscarriage, employment status, parity, desire for pregnancy(wanted pregnancy), religiosity, partner education, partner age and presence of the father of the child

**Table 2:** Factors associated with adequate frequency of ANC attendance (≥4 visits) among women aged 18–44 years in last pregnancy (*N* = 829)

Variables	Rural *(N = 363)*		Urban *(N = 466)*	
	Odds Ratio^a^ (95 % CI)	*p*-value	Odds Ratio^a^ (95 % CI)	*p*-value
Married	2.1 (0.9–4.6)	0.068	1.6 (0.9–2.7)	0.129
Previous miscarriage	0.4 (0.2–0.8)	0.009**	-	-
More than one child	0.8 (0.4–1.4)	0.368	-	-
Wanted pregnancy	4.2 (1.9–9.3)	0.001**	-	-
High Religiosity	0.7 (0.4–1.5)	0.361	1.3 (0.7–2.5)	0.333
Partner with higher	0.7 (0.3–1.5)	0.365	1.1 (0.6–2.0)	0.734
education Older Partner Age	0.9 (0.4–1.9)	0.747	-	-
Father of child present	3.1 (1.0–9.0)	0.042*	-	-

**p < 0.01 *p < 0.05

^a^Odds ratio adjusted for clustering. Final model for Rural (WC) site: marital status; previous miscarriage, parity, desire for pregnancy(wanted pregnancy), religiosity, partner education, partner age and presence of the father of the child. Final model for Urban (Tshwane) site: marital status; religiosity and partner education
  Results of multiple logistic regressions of frequency of ANC attendance did not generate any statistically significant associations for the urban site.

## Discussion

Our study identified psychosocial factors associated with early ANC initiation and frequency of ANC attendance in two contrasting sites, a rural farming area with a predominantly coloured population and an urban township area with a predominantly black population. The population of the urban site is broadly similar to other urban township populations in other provinces of South Africa. However, the population in the rural site is dissimilar to other rural farming populations in other provinces in South Africa because of particular historical, cultural and political factors shaping commercial farming in the Western Cape [31] and because the Western Cape is unique in that it is the only province in South Africa where farm workers are predominantly of coloured descent. Comparison of these two diverse sites thus enables an exploration of how psychosocial factors are associated with pregnancy outcomes across two different geographical and social contexts.

Individual level psychosocial factors such as pregnancy desire (wanted/unwanted pregnancy), religious orientation as well as partner characteristics (age and level of education) were more likely to be associated with timing of ANC initiation than the community level measures such as social support and social capital where the associations with ANC initiation were not significant.

### Rural site

Our findings are in agreement with other studies which also found that having a husband with low levels of education can be a barrier to access to antenatal care services [7]. However, having a poor relationship with the baby’s father, despite his presence during the pregnancy, may also negatively influence antenatal care attendance [30]. Our findings also resonate with those of other studies which found that late attendance to ANC was associated with factors such as unstable relationships with the unborn baby’s father, having a lower educated and employment status [32, 33].

Maternal health in many low resource countries including South Africa is still viewed as a woman’s issue due to socially constructed gender roles and men are generally not actively involved in reproductive health issues [34]. The findings of this study indicate that having the father of the child present in the woman’s life during pregnancy increases the odds of frequent ANC attendance three fold and highlight a need to involve male partners in reproductive health issues including family planning and maternal health care.

The findings on the associations of ANC attendance with religiosity suggest that high religiosity is a barrier to early ANC initiation for rural women. Studies in South Africa and Zimbabwe have revealed that culture and religion influence antenatal care attendance and that practices attached to these beliefs tend to delay ANC initiation [35, 36]. Women with high religiosity may feel less in need of assistance in pregnancy because they trust in their cultural and religious practices in ensuring a healthy pregnancy experience and positive health outcomes over presenting to a health facility to initiate antenatal care in the first trimester [36].

Interventions to reduce prevalence of late ANC booking and inadequate ANC attendance in rural communities should engage religious leaders with the help of community leaders as mediators and aim to address any misconceptions about ANC and educate them on the importance and benefits of early ANC initiation and adequate ANC attendance. Religious leaders are often regarded as “wise” and tend to have influence over the behaviour of other community members.

### Urban site

Late ANC initiation by unemployed women may be attributed to inability to meet the costs associated with the ANC visit such as transport and food, despite the actual health service being provided free of charge. Transport has been identified as the biggest cost of ANC in the South African context [37]. Employed women may also have access to health insurance and seek private health care services and better quality of care compared to unemployed women who rely on the public health facilities. In the public health sector in general, increasing the number of ANC clinics that are physically detached from the main health facility and introducing mobile ANC could increase utilization of these services and motivate early ANC initiation and adequate attendance. These measures could address women’s lack of access to antenatal clinics and eliminate travel costs associated with visits to the clinics.

Pregnancy desire is an important factor in determining timing of ANC initiation and our results confirm findings in previous studies [6, 28, 38]. It may therefore be useful to focus on increasing contraceptive uptake and family planning services in these populations [39]. Health education programmes should be tailored to the different social contexts and age categories of women, to encourage free interaction and be aimed at informing and educating all women on the different family planning options available to prevent unintended pregnancy and make these options easily accessible to all women.

Interestingly, fewer psychosocial variables examined were significantly associated with ANC attendance than would be expected. For example social support, social capital, self-esteem and mental health status were not significantly associated with outcome variables as has been found in the literature [16, 24, 30]. These findings can be attributed to reduced power of the study arising from how the variables were originally measured, the adaptations (or lack thereof) made to the instruments used and the questionnaire design. Because the original study from which the data was derived was based on sample size calculations for alcohol exposed pregnancies [17] the smaller sample size arising from the strict inclusion criteria for this study may have reduced the power of the study. Increasing the sample size would address these limitations and ensure that the study is sufficiently powered. Also, non-response (26 % and 17 % for urban and rural sites respectively) could have led to failure to capture those women with poor mental health and low self-esteem etc., resulting in the current findings. Other psychosocial factors associated with late initiation of antenatal care and infrequent ANC visits which were not considered in the current study include abortion contemplation and delayed diagnosis of the pregnancy [6].

When all factors are considered together, the results suggest that a rural coloured woman who initiated ANC early and attended ANC adequately tended to be a married woman with no previous miscarriage, who was not highly religious and had desired to fall pregnant when she conceived (wanted pregnancy) with a partner with higher education (completed at least Grade 9) and who was the father of the child she was carrying during that pregnancy. A black woman from the urban site who initiated ANC early and adequately attends ANC visits was employed and had desired to fall pregnant when she conceived (wanted pregnancy). The fact that the patterns of associations differed in these two populations is consistent with our a priori expectation that the social and cultural context of these two populations are very different [17]. Because the data used in this study came from a cross-sectional survey, our findings cannot confirm causal relationships because of a lack of temporality, but suggest associations between the psychosocial factors and outcomes which could be usefully explored in longitudinal studies.

### Limitations

The use of a pre-existing data set limited the study to the questionnaire design which was used in the data collection. Recall bias may also have affected the responses as women had to remember when they went for their first ANC visit and the frequency of their attendance of ANC clinics during their last pregnancy. For example, gestational age at first ANC visit was originally collected in months as this was easier for women to recall and then converted to weeks. This could have resulted in misclassification of early/ late attendance for example, a woman who reported gestational age of 4 months at first ANC visit who could have been either 4 months and 3 weeks pregnant or exactly 4 months pregnant but would be classified as an early attender (16 weeks) regardless.

Although frequency of ANC attendance was explored, our study did not examine frequency of timely ANC attendance. This was not an objective of the original study but may also have impacted the findings as ‘adequate attendance’ defined in the current study was based on the number of ANC visits and not whether the visits were timely, as per the WHO focused ANC model.

We acknowledge that there is potential bias associated with self-report on ANC visits as the participants were not required to present their ANC cards during the interviews to confirm their booking and ANC attendance. Questions on alcohol and drug use during the previous pregnancy may not have been well received as sensitive questions of this nature are usually prone to social desirability bias. Dichotomisation of social support and social capital variables to either ‘weak’ or ‘strong’ could have led to an underestimation of the extent of variation and probably under-estimated any effects identified in analyses.

Due to the lack of local South African studies using the same instruments used in our study to measure some of the psychosocial factors we could not compare results or ensure appropriate adjustments had been made, for example; and measures based on the instrument adapted from Martin and colleagues [21] to measure social capital are lacking within the South African context.

## Conclusions

These findings are useful to inform planning of health education programmes on antenatal care and for the implementation of holistic interventions aimed at improving ANC adherence (early initiation and adequate attendance) for better health outcomes for women in South Africa as timing of initiation of antenatal care in previous pregnancies may influence timing of initiation in subsequent pregnancies.

Whilst the majority of women from the rural and urban sites attended ANC frequently, most women initiated ANC late with less than 50 % initiating at or before 16 weeks gestational age. In a country faced with a high HIV/AIDS disease burden, early ANC initiation is crucial as it facilitates early HIV testing and subsequently, early ART initiation for eligible women [5].

## Additional files

Additional file 1:
**Bivariate analyses of associations between all demographic and all psychosocial factors examined against early ANC initiation among women aged 18–44 years who reported ever being pregnant (N=829): Comparison for early ANC initiation.** (DOCX 32 kb) Additional file 2:
**Bivariate analyses of associations between all demographic and all psychosocial factors examined against frequency of ANC attendance among women aged 18–44 years who reported ever being pregnant (N=829): Comparison for adequate ANC attendance.** (DOCX 32 kb)

## Abbreviations

AIC: Aikakes information criterion
ANC: antenatal care
AUDIT: alcohol use disorders identification test
BANC: basic antenatal care
LMIC: low and middle income countries
MMR: maternal mortality ratio
MOS: rand medical outcomes
WHO: World Health Organisation
