# Supplementary material for: Psychosocial factors associated with early initiation and frequency of antenatal care (ANC) visits in a rural and urban setting in South Africa: a cross-sectional survey
Source: BMC Pregnancy Childbirth. 2016 Jan 25;16:18. doi: 10.1186/s12884-016-0807-1 (PMC4727269; doi:10.1186/s12884-016-0807-1)
Supplement: Additional file 1: — Bivariate analyses of associations between all demographic and all psychosocial factors examined against early ANC initiation among women aged 18–44 years who reported ever being pregnant (N=829): Comparison for early ANC initiation. (DOCX 32 kb) [file 12884_2016_807_MOESM1_ESM.docx]

Table S1: Bivariate associations between individual demographic and psychosocial factors with early ANC initiation among the women (N=829).

| **Urban site** | | **N**  **(% Early ANC initiation)** | **OR**  **(95%CI)** | **Rural site** | | **N**  **(% Early ANC initiation)** | **OR**  **(95%CI)** |
| --- | --- | --- | --- | --- | --- | --- | --- |
| **Age**  18-24  25-34  35-44 | n=75(16%)  n=203(44%)  n=188(40%) | 27 (36)  86 (42)  103 (55) | -  1.3(0.8-2.3)  2.2(1.2-3.7) | **Age**  18-24  25-34  35-44 | n=75(21%)  n=149(39%)  n=139(38%) | 31 (41)  64 (43)  70 (50) | -  1.1(0.6-1.9)  1.4(0.8-2.5) |
| **Marital Status**  Married  Not married | n=120(27%)  n=326(73%) | 69 (58)  134 (41) | 1.9(1.3-3.0) | **Marital Status**  Married  Not married | n=119(33%)  n=244(67%) | 64 (54)  101 (41) | 1.7(1.1-2.6) |
| **Employed**  Yes  No | n=211(45%)  n=255(55%) | 113 (54)  103 (40) | 1.7(1.2-2.5) | **Employed**  Yes  No | n=295(81%)  n=68(19%) | 131 (44)  34 (50) | 0.8(0.5-1.4) |
| **Education**  Below Grade 9  Grade 9 and above | n=426(91%)  n=40(9%) | 196 (46)  20 (50) | 1.2(0.6-2.2) | **Education**  Below Grade 9  Grade 9 and above | n=262(72%)  n=101(28%) | 117 (45)  48 (48) | 1.1(0.7-1.8) |
| **Race**  Black/African  Coloured | n=378(87%)  n=56(13%) | 166 (44)  28 (50) | 1.3(0.7-2.2) | **Race**  Black/African  Coloured | n=30(8%)  n=329(92%) | 11 (37)  152 (46) | 1.5(0.7-3.2) |
| **Miscarriage**  Never  Previous miscarriage | n=363(78%)  n=103(22%) | 160 (44)  56 (54) | 1.5(1.0-2.4) | **Miscarriage**  Never  At least 1 miscarriage | n=283(78%)  n= 79(22%) | 125 (44)  39 (49) | 1.2(0.8-2.0) |
| **Parity**  0-1 child  More than one child | n=168(36%)  n=298(64%) | 72 (48)  144 (43) | 1.3(0.9-1.8) | **Parity**  0-1 child  More than one child | n=123(34%)  n=240(66%) | 50 (41)  115 (48) | 1.3(0.9-2.1) |
| **Desire**  Yes  No | n=321(69%)  n=145(31%) | 161 (50)  55 (38) | 1.7(1.1-2.5) | **Desire**  Yes  No | n=290(80%)  n=73(20%) | 142 (49)  23 (32) | 2.1(1.2-3.6) |
| **Social Support**  *Positive social interaction*  Strong  Weak  *Affectional support*  Strong  Weak  *Emotional support*  Strong  Weak | n=190(41%)  n=276(59%)  n=192(41%)  n=274(59%)  n=111(24%)  n=355(76%) | 95 (50)  121 (44)  97 (51)  119 (44)  51 (46)  165 (46) | 1.3(0.9-1.9)  1.3(0.9-1.9)  1.0(0.6-1.5) | **Social Support**  *Positive social interaction*  Strong  Weak  *Affectional support*  Strong  Weak  *Emotional support*  Strong  Weak | n=139(38%)  n=224(62%)  n=155(43%)  n=208(57%)  n=84 23%)  n=279(77%) | 72 (52)  93 (42)  78 (50)  87 (42)  46 (50)  119 (42) | 1.5(1.0-2.3)  1.4(0.9-2.1)  1.6(1.0-2.7) |
| **Social Capital**  Strong  Weak | n=121(26%)  n=345(74%) | 59 (49)  157 (46) | 1.1(0.8-1.7) | **Social Capital**  Strong  Weak | n=146(40%)  n=217(60%) | 62 (43)  103 (48) | 0.8(0.5-1.3) |
| **Mental Health**  High  Low | n=133(29%)  n=333(71%) | 64 (48)  152 (46) | 1.1(0.7-1.7) | **Mental Health**  High  Low | n=270(74%)  n=93(26%) | 119 (44)  46 (49) | 0.8(0.5-1.3) |
| **Cultural Influences**  *Male entitlement*  Agree  Disagree  *Wrong to not have children*  Agree  Disagree  *Female Worth*  Agree  Disagree  *Male Worth*  Agree  Disagree | n=176(38%)  n=289(62%)  n=190(41%)  n=274(59%)  n=310(67%)  n=155(33%)  n=297(64%)  n=168(36%) | 78 (44)  138 (48)  92 (48)  124 (45)  145 (47)  71 (46)  138 (47)  78 (47) | 0.9(0.6-1.3)  1.1(0.8-1.7)  1.0(0.7-1.5)  1.0(0.7-1.5) | **Cultural Influences**  *Male entitlement*  Agree  Disagree  *Wrong to not have children*  Agree  Disagree  *Female Worth*  Agree  Disagree  *Male Worth*  Agree  Disagree | n=88(24%)  n=275(76%)  n=120(33%)  n=243(67%)  n=306(84%)  n=57(16%)  n=299(82%)  n=64(18%) | 42 (48)  123 (45)  114 43)  51 (47)  143 (47)  22 (39)  139 (46)  26 (41) | 1.1(0.7-1.8)  0.8(0.5-1.3)  1.4(0.8-2.5)  1.3(0.7-2.2) |
| **Self Esteem**  High  Low | n=116(25%)  n=350(75%) | 54 (47)  162 (46) | 1.0(0.7-1.5) | **Self Esteem**  High  Low | n=93(26%)  n=270(74%) | 43 (46)  122 (45) | 1.0(0.7-1.7) |
| **Religious Orientation**  Highly religious  Not religious | n=131(28%)  n=355(76%) | 69 (53)  146 (44) | 1.4(1.0-2.1) | **Religious Orientation**  Highly religious  Not religious | n=122(31%)  n=247(69%) | 41 (37)  123 (50) | 0.6(0.4-0.9) |
| **Partner Characteristics**  *Age*  Older than 29yrs  Younger than 29yrs  *Employed*  Yes  No  *Education*  Below Grade 9  Grade 9 and above | n=419 (90%)  n=47 (10%)  n=319 (68%)  n=147 (32%)  n=91 (21%)  n=342(79%) | 202 (48)  14 (30)  165 (52)  51 (35)  44 (48)  157 (46) | 2.2(1.1-4.2)  2.0(1.4-3.0)  0.9(0.6-1.4) | **Partner Characteristics**  *Age*  Older than 29yrs  Younger than 29yrs  *Employed*  Yes  No  *Education*  Below Grade 9  Grade 9 and above | n=182(50%)  n=181(50%)  n=305(84%)  n=58(16%)  n=221(68%)  n=103(32%) | 87 (48)  78 (43)  143 (47)  22 (38)  91 (41)  56 (54) | 1.2(0.8-1.8)  1.4(0.8-2.6)  1.5(0.9-2.5) |
| *Father of the child present*  Yes  No | n=418 (90%)  n=48 (10%) | 17 (48)  199 (35) | 1.7(0.9-3.1) | *Father of the child present*  Yes  No | n=327(90%)  n=36(10%) | 154 (47)  11 (31) | 2.0(1.0-4.3) |
| **Substance Use**  *Ever smoked*  Yes  No  *Ever drank alcohol*  Yes  No    *AUDIT*  Score$\geq8$  Score$<8$ | n=86(19%)  n=378(81%)  n=177(38%)  n=288(62%)  n=31(7%)  n=435(93%) | 43 (50)  173 (46)  86 (49)  129 (45)  14 (45)  202 (46) | 1.2(0.7-1.9)  1.2(0.8-1.7)  0.9(0.5-2.0) | **Substance Use**  *Ever smoked*  Yes  No  *Ever drank alcohol*  Yes  No  *AUDIT*  Score$\geq8$  Score$<8$ | n=104(29%)  n=259(71%)  n=265(73%)  n=98(27%)  n=318(88%)  n=45(12%) | 110 (42)  55 (53)  115 (43)  50 (51)  140 (44)  25 (56) | 0.7(0.4-1.0)  0.7(0.5-1.2)  0.6(0.3-1.2) |

Note: Bivariate analyses of associations between individual demographic and psychosocial factors with early initiation among the women aged 18-44 years who reported ever being pregnant (N=829): comparison for early booking.
