# Supplementary material for: Psychosocial factors associated with early initiation and frequency of antenatal care (ANC) visits in a rural and urban setting in South Africa: a cross-sectional survey
Source: BMC Pregnancy Childbirth. 2016 Jan 25;16:18. doi: 10.1186/s12884-016-0807-1 (PMC4727269; doi:10.1186/s12884-016-0807-1)
Supplement: Additional file 2: — Bivariate analyses of associations between all demographic and all psychosocial factors examined against frequency of ANC attendance among women aged 18–44 years who reported ever being pregnant (N=829): Comparison for adequate ANC attendance. (DOCX 32 kb) [file 12884_2016_807_MOESM2_ESM.docx]

Table S2: Bivariate associations between individual demographic and psychosocial factors with adequate ANC attendance among the women (N=829).

| **Urban site** | | **N**  **(% Adequate ANC attendance)** | **OR**  **(95%CI)** | **Rural site** | | **N**  **(% Adequate ANC attendance)** | **OR**  **(95%CI)** |
| --- | --- | --- | --- | --- | --- | --- | --- |
| **Age**  18-24  25-34  35-44 | n=75(16%)  n=203(44%)  n=188(40%) | 63 (84)  165 (81)  165 (88) | -  0.8(0.4-1.7)  1.4(0.6-2.9) | **Age**  18-24  25-34  35-44 | n=75(21%)  n=149(39%)  n=139(38%) | 56 (75)  112 (75)  116 (83) | 1.0(0.5-2.0)  1.7(0.9-3.4) |
| **Marital Status**  Married  Not married | n=120(27%)  n=326(73%) | 107 (89)  268 (82) | 1.8(0.9-3.4) | **Marital Status**  Married  Not married | n=119(33%)  n=244(67%) | 101 (85)  183 (75) | 1.9(1.1-3.3) |
| **Employed**  Yes  No | n=211(45%)  n=255(55%) | 174 (83)  219 (86) | 0.8(0.5-1.3) | **Employed**  Yes  No | n=295(81%)  n=68(19%) | 229 (78)  55 (81) | 0.8(0.4-1.6) |
| **Education**  Below Grade 9  Grade 9 and above | n=426(91%)  n=40(9%) | 360 (85)  33 (83) | 0.9(0.4-2.0) | **Education**  Below Grade 9  Grade 9 and above | n=262(72%)  n=101(28%) | 209(80)  75 (75) | 0.7(0.4-1.3) |
| **Race**  Black/African  Coloured | n=378(87%)  n=56(13%) | 321 (85)  47 (84) | 0.9(0.4-2.0) | **Race**  Black/African  Coloured | n=30(8%)  n=329(92%) | 22 (74)  258 (78) | 1.3(0.6-3.1) |
| **Miscarriage**  Never  Previous miscarriage | n=363(78%)  n=103(22%) | 304 (84)  89 (86) | 1.2(0.7-2.3) | **Miscarriage**  Never  At least 1 miscarriage | n=283(78%)  n= 79(22%) | 228 (81)  55 (70) | 0.6(0.3-1.0) |
| **Parity**  0-1 child  More than one child | n=168(36%)  n=298(64%) | 136 (81)  257 (86) | 1.5(0.9-2.5) | **Parity**  0-1 child  More than one child | n=123(34%)  n=240(66%) | 94 (76)  190 (79) | 1.2(0.7-2.0) |
| **Desire**  Yes  No | n=321(69%)  n=145(31%) | 264 (82)  129 (89) | 0.6(0.3-1.0) | **Desire**  Yes  No | n=290(80%)  n=73(20%) | 241 (83)  43 (59) | 3.4(2.0-6.0) |
| **Social Support**  *Positive social interaction*  Strong  Weak  *Affectional support*  Strong  Weak  *Emotional support*  Strong  Weak | n=190(41%)  n=276(59%)  n=192(41%)  n=274(59%)  n=111(24%)  n=355(76%) | 166 (87)  227 (82)  169 (88)  224 (82)  97 (87)  296 (83) | 1.5(0.9-2.5)  1.6(1.0-2.8)  1.4(0.7-2.6) | **Social Support**  *Positive social interaction*  Strong  Weak  *Affectional support*  Strong  Weak  *Emotional support*  Strong  Weak | n=139(38%)  n=224(62%)  n=155(43%)  n=208(57%)  n=84 23%)  n=279(77%) | 111 (80)  173 (77)  119 (77)  165 (79)  68 (81)  216 (77) | 1.2(0.7-2.0)  0.9(0.5-1.4)  1.2(0.7-2.3) |
| **Social Capital**  Strong  Weak | n=121(26%)  n=345(74%) | 99 (82)  294 (85) | 0.8(0.5-1.4) | **Social Capital**  Strong  Weak | n=146(40%)  n=217(60%) | 110 (75)  174 (80) | 0.8(0.5-1.3) |
| **Mental Health**  High  Low | n=133(29%)  n=333(71%) | 115 (86)  278 (83) | 1.3(0.7-2.3) | **Mental Health**  High  Low | n=270(74%)  n=93(26%) | 213 (79)  71 (76) | 1.2(0.7-2.0) |
| **Cultural Influences**  *Male entitlement*  Agree  Disagree  *Wrong to not have children*  Agree  Disagree  *Female Worth*  Agree  Disagree  *Male Worth*  Agree  Disagree | n=176(38%)  n=289(62%)  n=190(41%)  n=274(59%)  n=310(67%)  n=155(33%)  n=297(64%)  n=168(36%) | 154 (88)  239 (83)  159 (84)  233 (85)  261 (84)  132 (85)  252 (85)  141 (84) | 1.5(0.9-2.5)  0.9(0.5-1.5)  0.9(0.5-1.6)  1.1(0.6-1.8) | **Cultural Influences**  *Male entitlement*  Agree  Disagree  *Wrong to not have children*  Agree  Disagree  *Female Worth*  Agree  Disagree  *Male Worth*  Agree  Disagree | n=88(24%)  n=275(76%)  n=120(33%)  n=243(67%)  n=306(84%)  n=57(16%)  n=299(82%)  n=64(18%) | 70 (80)  214 (78)  90 (75)  194 (80)  234 (76)  50 (88)  228 (76)  56 (88) | 1.1(0.6-2.0)  0.8(0.5-1.3)  0.5(0.2-1.1)  0.5(0.2-1.0) |
| **Self Esteem**  High  Low | n=116(25%)  n=350(75%) | 98 (85)  295 (84) | 1.0(0.6-1.8) | **Self Esteem**  High  Low | n=93(26%)  n=270(74%) | 76 (82)  208 (77) | 1.3(0.7-2.4) |
| **Religious Orientation**  Highly religious  Not religious | n=131(28%)  n=355(76%) | 116 (89)  275 (83) | 1.6(0.9-3.0) | **Religious Orientation**  Highly religious  Not religious | n=122(31%)  n=247(69%) | 84 (75)  196 (79) | 0.8(0.5-1.3) |
| **Partner Characteristics**  *Age*  Older than 29yrs  Younger than 29yrs  *Employed*  Yes  No  *Education*  Below Grade 9  Grade 9 and above | n=419 (90%)  n=47 (10%)  n=319 (68%)  n=147 (32%)  n=91 (21%)  n=342(79%) | 354 (85)  39 (83)  275 (85)  118 (80)  75 (84)  289 (85) | 1.1(0.5-2.5)  1.5(0.9-2.6)  0.9(0.5-1.6) | **Partner Characteristics**  *Age*  Older than 29yrs  Younger than 29yrs  *Employed*  Yes  No  *Education*  Below Grade 9  Grade 9 and above | n=182(50%)  n=181(50%)  n=305(84%)  n=58(16%)  n=221(68%)  n=103(32%) | 142 (78)  142 (78)  251 (82)  33 (57)  170 (80)  80 (78) | 1.0(0.6-1.6)  3.5(1.9-6.4)  1.1(0.6-2.2) |
| *Father of the child present*  Yes  No | n=418 (90%)  n=48 (10%) | 354 (85)  39 (81) | 1.3(0.6-2.8) | *Father of the child present*  Yes  No | n=327(90%)  n=36(10%) | 264 (81)  20 (56) | 3.4(1.6-6.8) |
| **Substance Use**  *Ever smoked*  Yes  No  *Ever drank alcohol*  Yes  No    *AUDIT*  Score$\geq8$  Score$<8$ | n=86(19%)  n=378(81%)  n=177(38%)  n=288(62%)  n=31(7%)  n=435(93%) | 71 (83)  321 (85)  142 (80)  250 (87)  27 (87)  366 (84) | 0.8(0.5-1.6)  0.6(0.4-1.0)  1.3(0.4-3.8) | **Substance Use**  *Ever smoked*  Yes  No  *Ever drank alcohol*  Yes  No  *AUDIT*  Score$\geq8$  Score$<8$ | n=104(29%)  n=259(71%)  n=265(73%)  n=98(27%)  n=318(88%)  n=45(12%) | 198 (76)  86 (83)  208 (78)  76 (78)  249 (78)  35 (78) | 0.7(0.4-1.2)  1.1(0.6-1.9)  1.0(0.5-2.2) |

Note: Bivariate analyses of associations between individual demographic and psychosocial factors with adequate ANC attendance among women aged 18-44 years who reported ever being pregnant (N=829): comparison for adequate attendance.
